# Quantification of Motility in *Bacillus subtilis* at Temperatures Up to 84°C Using a Submersible Volumetric Microscope and Automated Tracking

**DOI:** 10.3389/fmicb.2022.836808

**Published:** 2022-04-21

**Authors:** Megan M. Dubay, Nikki Johnston, Mark Wronkiewicz, Jake Lee, Christian A. Lindensmith, Jay L. Nadeau

**Affiliations:** ^1^Department of Physics, Portland State University, Portland, OR, United States; ^2^Jet Propulsion Laboratory, California Institute of Technology, Pasadena, CA, United States

**Keywords:** *Bacillus subtilis*, bacterial motility, temperature effects, heat shock, holographic microscopy, thermophile, tracking

## Abstract

We describe a system for high-temperature investigations of bacterial motility using a digital holographic microscope completely submerged in heated water. Temperatures above 90°C could be achieved, with a constant 5°C offset between the sample temperature and the surrounding water bath. Using this system, we observed active motility in *Bacillus subtilis* up to 66°C. As temperatures rose, most cells became immobilized on the surface, but a fraction of cells remained highly motile at distances of >100 μm above the surface. Suspended non-motile cells showed Brownian motion that scaled consistently with temperature and viscosity. A novel open-source automated tracking package was used to obtain 2D tracks of motile cells and quantify motility parameters, showing that swimming speed increased with temperature until ∼40°C, then plateaued. These findings are consistent with the observed heterogeneity of *B. subtilis* populations, and represent the highest reported temperature for swimming in this species. This technique is a simple, low-cost method for quantifying motility at high temperatures and could be useful for investigation of many different cell types, including thermophilic archaea.

## Introduction

The effects of elevated temperatures on bacterial motility have not been fully explored. There are both physical and physiological effects of temperature on flagellar swimming at low Reynolds number. Both viscosity and temperature contribute to the Stokes-Einstein equation for the diffusion coefficient, *D*


(1) $$D=\frac{k_{B}T}{6\pi \eta r}$$

where *k*_*B*_ is Boltzmann’s constant, *T* is the absolute temperature, η is the dynamic viscosity of the medium, and *r* is the radius of the diffusing particle. Water shows a dramatic decrease in dynamic viscosity with temperature, described by the equation


(2) $$\eta \left(T\right)=Ae^{B/\left(T-C\right)},$$

where A = 2.414 × 10^–5^ Pa•s, B = 247.8 K, and C = 140 K. This results in values ranging from 1.002 mPa s at 20°C to 0.315 mPa s at 90°C. As a result, there is a significant change in the rate of Brownian motion of cells at elevated temperatures: as a simple example, 0.30 μm^2^/s at 33°C for a 1 μm diameter cell, and 0.86 μm^2^/s for the same cell at 91°C.

The effects of viscosity on active swimming are less clear. The drag force is proportional to η and thus it would be expected that swimming speeds would increase with decreased viscosity, but instead a surprising decrease in bacterial swimming speeds with decreased viscosity is seen in polymer solutions. Several papers have suggested that this is due to microstructure of the polymer (Magariyama and Kudo, 2002; Zottl and Yeomans, 2019). In ordinary aqueous solution, a roughly linear increase in swimming speed with temperatures up to 50°C has been reported for *Escherichia coli* (Maeda et al., 1976) and for multiple other strains representing polar, bipolar, and peritrichous flagellar arrangements (Schneider and Doetsch, 1977). One study reported a linear increase in *E. coli* swimming speed with temperature up to 40°C in medium supplemented with L-serine; in the absence of supplementation, speeds increased only up to 30°C and decreased thereafter (Demir and Salman, 2012). This linear relationship is related to increased flagellar rotation rates at high temperatures as well as altered viscosities both inside and outside the cell and has been modeled semi-empirically using a large number of available motility datasets. Speed is generally assumed to be directly proportional to flagellar rotation rate; though this is not true in all datasets, a positive correlation is always present (Humphries, 2013).

As a simple model, the force on a swimming cell may be approximated as the sum of the flagellar force *F* and the velocity-dependent drag force *Dv*,


(3) F=ma=F-Dv,

where D is the drag coefficient given in Eq. (1). Although models predict a viscosity dependence, recent studies have found that F is independent of viscosity (Armstrong et al., 2020). This equation of motion yields a terminal velocity of


(4) $$v_{\infty }=\frac{F}{D}\propto \frac{F}{\eta },$$

where the terminal velocity should be observed during a long run once the cell is no longer accelerating. The time-averaged velocity over a long run should thus approximate *v*_∞_.

None of these models take into account the upper limits of possible motility of different strains resulting from protein denaturation or general organism stress. Microorganisms show complex heat shock responses, and the expression and maintenance of flagella can be affected by genes related to heat stress. Motility is a complex phenotype under tight regulation in all microorganisms that express it. *Bacillus subtilis* is a model organism for which regulation of motility genes (Mukherjee and Kearns, 2014) and heat shock responses (Schumann, 2003) have been well studied. There is a strict dependence upon FlgN for motility in this species (Cairns et al., 2014). Flagellar synthesis is affected by a number of the genes involved in the heat shock response. As temperature increases, proteins denature, and protein degradation systems clear the damaged proteins. The ClpCP complex in *Bacillus subtilis* degrades stress-damaged proteins as well as taking part in regulatory degradation. It also influences motility by both direct and indirect mechanisms. The absence of Clp proteases results in defective motility, likely due to accumulation of Spx, which suppresses flagellar gene expression. Cells under stress do not necessarily lose motility immediately via this mechanism, since existing flagella are not affected, rather the production of new flagella (Moliere et al., 2016).

The heat shock responses of *B. subtilis* allow it to grow to at least 53°C (Warth, 1978). Much has been written about the mechanisms of thermotolerance in this species. The sigma factor σ^B^ provides non-specific stress resistance, which includes thermotolerance. Its activation induces the expression of ∼200 target genes (Hecker et al., 1996; Nannapaneni et al., 2012; Young et al., 2013). Cells do not need to be exposed to heat stress in order to gain thermotolerance via this mechanism. In addition, there are specific heat-shock responses that turn on under conditions of mild heat stress, leading to protection from otherwise lethal temperatures. At least three classes of genes are heat-shock inducible.

Because of the complexity of both the heat shock response and regulation of motility genes, it is difficult to predict the effects of high temperature on swimming motility. Most studies focus on growth rather than persistence of swimming. The persistence of the motility phenotype under conditions of heat stress has been little explored, largely due to technical difficulties of imaging and image analysis as temperatures rise. One study reported that convection currents made tracking difficult above 40°C (Riekeles et al., 2021). In addition, most heated stages can only achieve temperatures of 55–60°C. We hypothesized here that some fraction of *B. subtilis* cells would be capable of active motility at temperatures above the maximum growth temperature, with significant variations consistent with the highly heterogeneous responses of this species to stress (Kearns and Losick, 2005; Lopez et al., 2009; Syvertsson et al., 2021).

A few studies have reported microscopic imaging systems able to reach temperatures up to the boiling point of water, but none fit our precise goals. A 1995 study of *Thermotoga maritima* used a capillary between two Peltier elements (Gluch et al., 1995). A more recent study reported an environmental chamber for microbial imaging that allows for pressure and temperature control (Nishiyama and Arai, 2017). For studying hyperthermophilic archaea, a “Sulfoscope” with a heated cap and stage was recently described where temperatures of 75°C were stably maintained; temperatures up to 90°C were achieved but led to significant evaporation (Pulschen et al., 2020). This design is appropriate for fluorescence microscopy and is especially valuable for imaging immobilized cells, a technique which was described in detail in the study using a semi-soft Gelrite pad. Air objectives were used. Another recent study attained higher resolution using oil-immersion objectives heated to 65°C, with the entire chamber heated to 75°C. Imaging was by differential interference contrast (DIC) (Charles-Orszag et al., 2021).

While high resolution is sometimes desirable, our goal was to create a simple, inexpensive system for imaging at high temperatures with a particular focus on tracking of microorganisms in a large volume of view. Thus, the goal was to maximize the depth of field with sufficient resolution to distinguish individual cells, but not subcellular structure. We use a custom holographic microscope entirely submerged in a heated water bath in order to examine motility at temperatures between 28 and 85°C, with capability of temperatures up to at least 95°C. The materials for the heated bath are inexpensive (<$200 USD in 2022) and a complete parts list is included for both the microscope and bath, with a total cost of <$7000 USD for the complete system. Because the system was designed for field use and no compound objective lenses are used, there are minimal effects of temperature on the instrument, even up to the boiling point.

With this system, cultures of *B. subtilis* were either heated slowly over a period of 4 h or exposed rapidly by submersion into a pre-heated bath. Individual cells were tracked, and velocities, accelerations, turn frequency, and correlation functions were all quantified using a custom open-source autonomous program. The program successfully controlled for thermal currents, classifying tracks into “motile” vs. “non-motile” based upon a defined algorithm. All active tracks were manually confirmed to correspond to cells. Active swimming was observed up to 66°C in some fraction of the cells; increasing numbers of immobile cells collected on the sample chamber surface as temperatures increased. Cells up to 90°C remained normal in morphology, without signs of sporulation. Dead cells showed little movement until temperatures >60°C, at which point convection currents became appreciable; however, these were readily distinguished from active motility by inspection as well as automated tracking tools. These techniques will be of interest to anyone exploring bacterial behavior at temperatures up to the boiling point of water.

## Materials and Methods

### Microscope and Temperature Control

**FIGURE 1:**
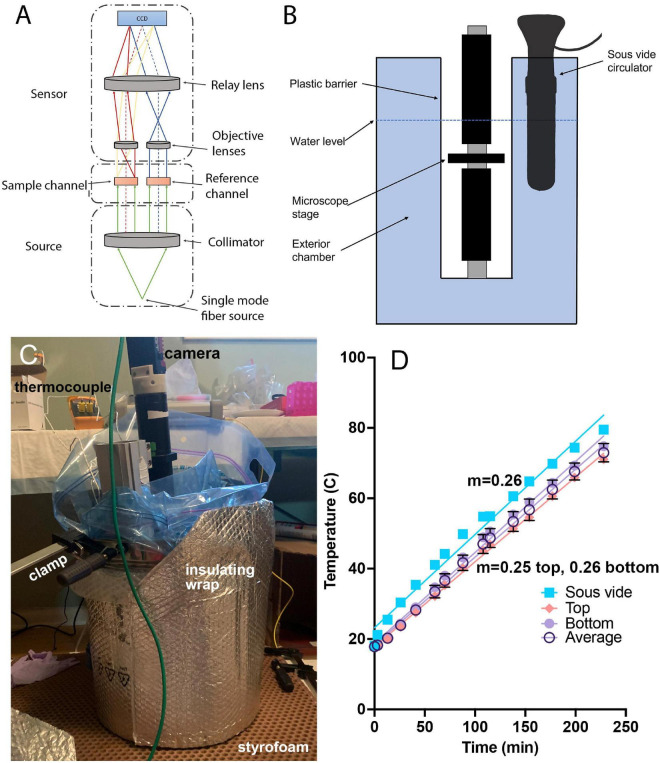
Temperature-controlled microscope setup. **(A)** Schematic of off-axis DHM used in these experiments. **(B)** The microscope was protected by a heavy-duty plastic bag and submerged in heated water to control the sample temperature. **(C)** Photograph of the complete setup showing the insulating materials necessary to attain the highest temperatures. The microscope is submerged into the pot with the sample chamber located several cm below the water surface. The insulation shown is required for achieving water and sample temperatures above ∼70°C. **(D)** Correspondence between the sous vide measured water temperature (verified with a laboratory thermometer) and thermocouple measurements of the sample chamber at top and bottom, along with the average between top and bottom. The slope of the temperature change was consistent between the bath and the chamber, with a nearly constant offset of 5°C. The lines are linear fits with slopes indicated.

The microscope used in this study was a common-path off-axis digital holographic microscope (DHM) as described previously (Wallace et al., 2015) (U.S. Patent US20160131882A1) (Figure 1A). Briefly, a single-mode laser (520 nm, Thorlabs, Newton, NJ) was collimated and passed through separate reference and sample channels held in a single plane to prevent mis-alignment. The objective lenses were simple achromats (numerical aperture 0.31, part number 47–689-INK, Edmund Optics, Barrington, NJ), yielding an effective magnification of ∼20x and XY spatial resolution of ∼1.0 μm. Since focusing is performed numerically with DHM, the sample stage was fixed in position with the in-focus plane *Z* = 0 at approximately the center of the sample chamber. The camera was a Prosilica 2460GT (Allied Vision, purchased from Edmund Optics) monochrome camera with a 5 MPixel, 3.45 μm pixel pitch format and 15 frames/s maximum frame rate, with acquisition windowed to 2,048 × 2,048 px. A higher frame rate (23.7 fps) can be achieved by substituting the newer Prosilica 2560GT without further modification of the system. Both recommended cameras use global shutter detector readout; rolling shutter readout cameras are not recommended unless careful consideration is taken in avoiding distortion of fringes during readout.

To control the temperature of the sample chamber, the entire microscope was placed into a stainless steel cooking pot (36 quart, Bayou Classic, sold through amazon.com) and protected by a 5-gallon, 4 mil thickness plastic bag (Ziploc brand or autoclave bag). The laser was kept out of the bath and coupled to the microscope through its single-mode fiber output. The camera was kept above the water level of the bath by the microscope tube. The pot was filled with water to a level above the top of the sample chamber, and the temperature of the water was gradually increased at a constant rate of 4.3°C/min using a sous vide circulator (Monoprice Model#: 121594, 800 W, 4 gallon capacity) (Figures 1B,C). The water temperature indicated on the circulator was confirmed using a laboratory thermometer (Fisherbrand). In order to raise the temperature above ∼80°C, it was necessary to insulate the pot with metallized bubble wrap (Figure 1C). A thermocouple (Gain Express K-Type, sold through amazon.com) was taped both above and below the chamber, in direct contact with the chamber, in an independent set of experiments to determine how the sample temperature corresponded to the water temperature; a nearly constant offset of 5°C was seen between the chamber and the water temperature, and this correction was applied to all reported chamber temperatures (Figure 1D). Supplementary Figures 1, 2 show the steps involved in setup, and Supplementary Datasheet 1 provides a parts list for the entire instrument with purchasing links. The costliest elements are the laser and camera.

Preliminary thermal testing of elements was performed by submerging individual parts of the setup into water in Ziploc bags and heating the water using the sous vide controller to its maximum temperature (just below boiling). In the case of the objective lens holder, because of its small size and low cost, it was placed into boiling water on the stovetop both with and without the lenses installed. The upper temperature limit of the microscope is set by the plastic used in the 3D-printed lens holder and the bonding of the sample chamber; substitutions of these components with higher temperature materials would enable higher temperature investigations. None of the components was tested above 100°C in this study.

### Samples and Chambers

*Bacillus subtilis* type strain (ATCC 6051) was obtained from the American Type Culture Collection, Manassas, VA. Stocks were maintained at −80°C and periodically streaked onto lysogeny broth (LB, Thermo Fisher Scientific, Pittsburgh, PA)-agar plates. 16–24 h before each experiment, single colonies were picked from plates, seeded into liquid LB, and incubated at 30°C with shaking to mid-log phase (OD ∼0.6 as measured on a Clariostar plate reader). After incubation, samples were diluted 1:1,000–1:100 into motility medium (10 mM phosphate buffer pH 7.4, 10 mM NaCl, 0.1 mM EDTA) and moved to custom sample chambers at 20 ± 2°C. To control for any possible non-biological motion at high temperatures, an additional set of experiments was performed using heat-killed *B. subtilis* cells at the same concentration. The cells were exposed to boiling water for 30 min, then pelleted and washed twice with motility medium before imaging.

**FIGURE 2:**
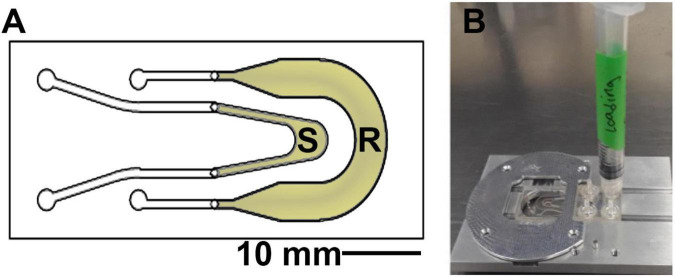
Sample chamber arrangement. **(A)** Schematic of the 1 mm deep chamber, showing the sample (S) and reference (R) channels that permit the off-axis DHM geometry. The volume of view of each snapshot is 365 μm × 365 μm × 1 mm in x, y, and z. **(B)** The chambers were loaded using a 1 mL syringe and placed onto the microscope stage at a fixed focus before immersion.

The sample chambers (product of Aline, Rancho Dominguez, CA) are composed of two channels, one for the sample and one as a reference channel which is filled with 0.9% saline solution or sterile growth medium (Figure 2). These are composed of two layers of optical quality glass with a middle acrylic layer forming the channels. Homemade chambers using microscope slides, coverslips, and adhesive material such as silicone or Teflon may also be used; for detailed instructions (see Supplementary Figures 3–5). The depth of the chamber is 1.0 mm, which allows for motility far away from the surfaces in order to eliminate surface influences on motility (Li et al., 2008;Giacche et al., 2010; Li et al., 2011). The sample was placed into the microscope before immersion and left throughout the duration of the heating. Replicate experiments (2–4) were performed on different days with independent *B. subtilis* cultures in order to confirm the reproducibility of the results. Cell density in the chambers varied from ∼10^5^ to 10^6^ cells/mL.

### Acquisition and Reconstruction of Holograms

Data were acquired using a custom, open-source software package, DHMx.^1^ Recordings were between 30 and 60 s long, with a maximum frame rate of 15 frames per second (fps). Recordings were obtained every ∼5°C until reaching a maximum sample temperature of 84°C. Additional tests were performed which subjected the bacteria to rapid increases in temperature. Samples were loaded into chambers at 20°C and submerged into pre-heated water at varying temperatures. For these “heat shock” experiments, recordings were obtained immediately after submersion as well as 2 and 10 min afterward. The 10 min datasets were chosen for analysis.

The holograms for each recording were either median subtracted (as we described previously Bedrossian et al., 2020) or frame-to-frame subtracted then reconstructed in amplitude using Fiji (ImageJ) (RRID:SCR_002285) (Schindelin et al., 2012). Reconstructions were performed using the angular spectrum method (Mann et al., 2005) implemented in a custom plug-in described in detail elsewhere (Cohoe et al., 2019) and available from our update site.^2^ The choice of frame-to-frame subtraction was necessary at temperatures >50°C in order to eliminate noise due to non-stationary cells on the chamber surface. The z thickness chosen for reconstruction ranged from 400 to 800 μm and varied somewhat among datasets depending upon the location of active cells. The resulting stacks were maximum projected in Z using Fiji to create a 2D time series of all of the cells of interest.

### High Resolution Light and Fluorescence Microscopy

In order to carefully evaluate cell morphology and possible presence of spores, cells were examined on an Olympus IX-71 inverted microscope with a 100x, NA = 1.4 oil immersion objective using either brightfield illumination or fluorescence illumination with a Hg lamp and a 450/50 nm excitation filter and 520 nm longpass (Chroma Technology, Bellows Falls, VT).

### Fraction Motile

Non-motile cells were difficult to count from images, especially at higher temperatures as cells began to cluster, so the fraction of motile cells was estimated as the number of motile cells per volume of view averaged over the length of the recording, divided by the total cell concentration (as measured by original OD divided by dilution factor; the relationship between OD and cell concentration was established using a hemocytometer). The instantaneous volume of view is 0.365 × 0.365 × 1 mm^3^, or 0.13 μL, corresponding to ∼100 cells/frame at 10^6^ cells/mL. The number of motile cells per frame was calculated using frame-to-frame subtracted projections so that non-motile cells did not interfere with the analysis. Fiji Analyze Particles^3^ was used to count the number of cells per frame.

### Tracking and Statistics

Bacteria were tracked using a custom software package, Holographic Examination for Life-like Motility (HELM),^4^ which was developed to autonomously detect, track, and characterize motile cells. HELM identifies pixel changes in sequential DHM images, tracks clusters of change as particle movement, and classifies particles as motile or non-motile based on their movement patterns. The pixel changes are computed by simple background subtraction of the median video image. Clusters of pixel changes are identified using DBSCAN. Tracks are then generated using the Linear Assignment Problem (LAP) tracker method (Jaqaman et al., 2008). With the spatiotemporal points, HELM computes approximately two dozen metrics that form a feature vector for each track. These include features like mean speed, mean turn angle, total track displacement, etc. A random forest classifier (Breiman, 2001) is then used to classify each track as motile or non-motile. The classifier was trained using manually labeled tracks from both prepared laboratory and field-acquired ocean water samples, both with and without fluid flow in the sample chamber. HELM can be used on raw, unreconstructed holograms or on 2D projections of reconstructed holograms. Analysis presented here was performed on projections of reconstructed holograms as described in section “Motility Analysis.”

HELM also generates multiple contextual products to support assessment of a DHM recording. One of these, the Motion History Image (MHI), summarizes a full video in one image by color mapping each pixel to the time index of largest intensity change. The MHI image allows a rapid understanding of how many particles were present in the recording as well as the presence and characteristics of potential motile organisms.

Diffusion coefficients were measured using NanoTrackJ (Wagner et al., 2014). A video of at least 30 s containing at least 10 trackable particles was analyzed using the Maxima and Gaussian Fit center estimator and the covariance diffusion coefficient estimator. Parameters used were: minimum estimated particle size, 20 pixels; minimum number of steps per track, 5; pixel size, 178 nm; and frame rate 15 frames/s.

Statistical analysis of HELM outputs and graphing were performed using Prism 9 (GraphPad Software, San Diego, CA). Statistical relevance was estimated using the ANOVA package in Prism after ensuring that distributions were Gaussian. The correlation matrix was computed using the Multivariable Analysis package in Prism.

## Results

### Microscope Function and Maintenance at High Temperatures

We tested all elements of the system for their ability to withstand temperatures up to 100°C. The DHM optics are robust, as they contain no compound objectives, adhesives, or other heat-sensitive elements. The 3D printed lens holder and objectives withstood boiling up to 30 min. The most sensitive element of the setup was the fiber optic cable. Under preliminary tests, the cable housing degraded, allowing light to escape, when the cable was heated to 90°C. This could be prevented by shielding the cable from the heat using an aluminum mini-box as shown in Supplementary Figure 1. On occasion, condensation would form on the sample chamber, objective lens, or collimating lens. This could be removed by wiping with lens paper. After multiple rounds of experiments or if image quality appeared poor, all of the lenses were removed and cleaned with 100% isopropanol. It was also important to keep the camera outside of the hot area to prevent condensation on the window.

### Morphology and Brownian Motion

**FIGURE 3:**
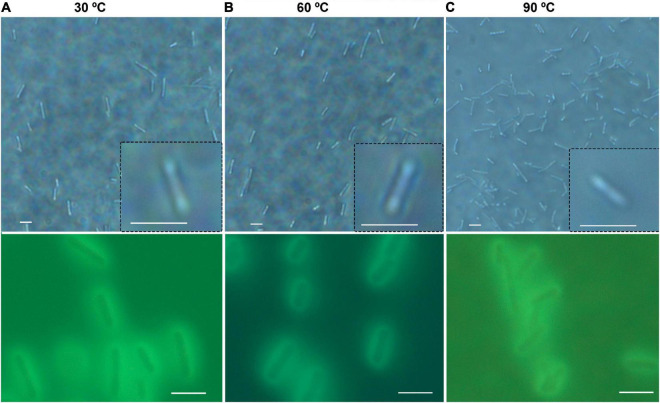
General appearance and size of *B. subtilis* at normal and elevated temperatures. Shown are phase contrast (top row) and autofluorescence (bottom row); scale bar = 5 μm. **(A)** 30°C. **(B)** 60°C. **(C)** 90°C.

**FIGURE 4:**
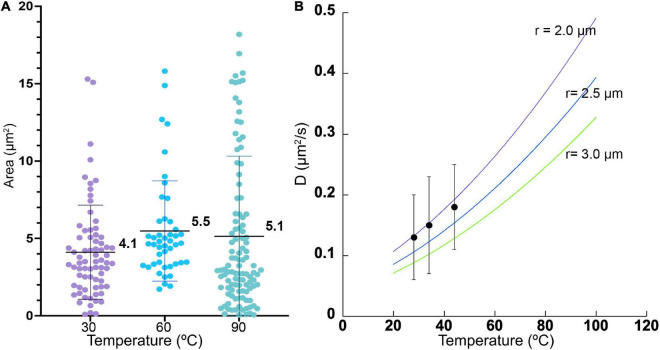
Cell sizes and diffusion coefficients with temperature. **(A)** Cell area as estimated by microscopy, shown as mean ± standard deviation. The differences in the means from one temperature to the next was not significant. **(B)** Measured diffusion coefficients at 3 temperatures (black dots), with measured standard deviations, compared with predicted values according to Eq. (1) for particle radii of 2.0, 2.5, and 3.0 μm.

There was no significant difference in size or overall morphology of motile cells as the temperature increased (Figures 3, 4A). Non-motile cells showed increased rates of Brownian motion consistent with scaling of diffusion rates with temperature (*k_*B*_T*) and dynamic viscosity of water (η). The Stokes-Einstein relation (Eq. 1) gave an excellent fit to the measured data for particle radius *r* = 2.1 μm (Figure 4B). This was consistent with the sizes measured by direct imaging, with a large amount of variability seen in both measured size and diffusion coefficient due to the presence of elongated and clustered cells. For temperatures higher than 44°C, Brownian motion was impossible to evaluate because of either thermal currents or large numbers of cells immobilized on the glass surface.

### Motility Analysis

**FIGURE 5:**
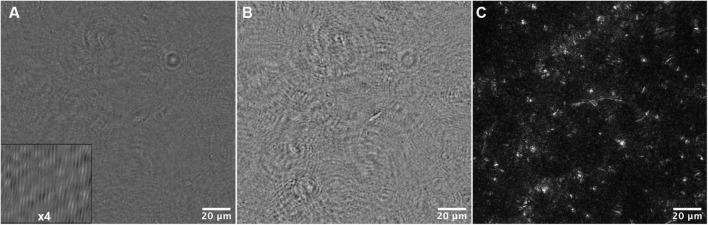
Data reconstruction and processing for tracking. **(A)** A portion of the field of view of a median-subtracted hologram from the 34°C dataset. The inset shows part of the image magnified 4x to show the fringes. **(B)** Amplitude reconstruction of the same field of view in **(A)** at +50 μm. **(C)** Maximum Z projection of the same field of view, representing reconstructions from −200 to +200 μm in steps of 10 μm.

**FIGURE 6:**
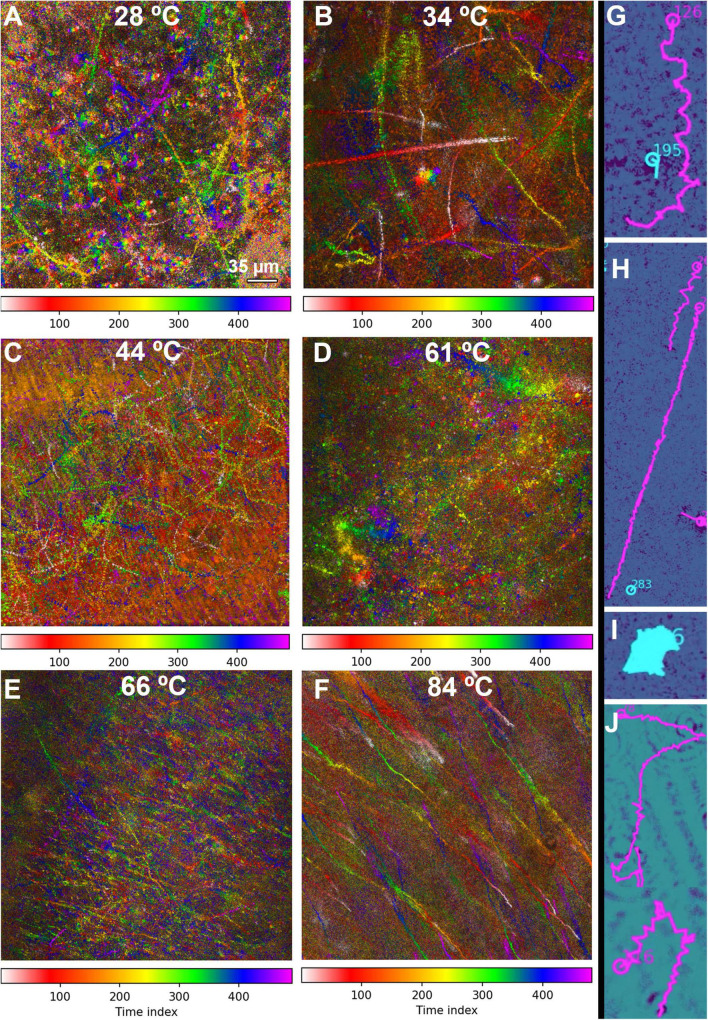
MHI analysis shows changes in motility patterns with increasing temperature and assists in identifying motile tracks. The tracks are time-coded, with the time index indicating frame number (15 frames/s). The scale bar applies to all panels. **(A–F)** Full field of view of all identified tracks in selected datasets. **(A)** 28°C, showing a distribution of motile and non-motile cells and distinct swimming patterns. **(B)** 34°C, showing increased motility and speed. **(C)** 44°C, showing nearly all cells motile at high speed. **(D)** 61°C, showing a reduction in the number of motile cells, but some swimming at high speed. **(E)** 66°C, showing a large amount of thermal drift with a few motile cells. **(F)** 84°C, with all motion due to thermal drift. **(G–J)** Selected examples of swimming types identified in the tracks. Magenta indicates tracks identified as motile by the software; tracks in cyan were identified as non-motile. **(G)** Helical swimming. **(H)** Long straight runs. **(I)** Circling or spinning. **(J)** Run and tumble.

Motility analysis was performed on reconstructions and projections of holograms. An example hologram is shown in Figure 5A, and a single-plane amplitude reconstruction in Figure 5B. Single plane reconstructions provided qualitative insight into cell morphologies, speeds, and fraction of motile cells; however, their signal to noise ratio was insufficient for automated tracking. Maximum projections through 40–80 Z planes, representing 400–800 μm sample depth (Figure 5C), permitted automated tracking to create 2D trajectories. Motion history images (MHIs, as described in “Materials and Methods”) were used to identify tracks of motile organisms from these projections (see Supplementary Video 1). Figure 6 shows time-coded MHIs for temperatures from 28 to 84°C for a selected set of experiments. The spacing between points on each track gives a rough estimate of cell speed. Where thermal drift was significant, motile cells could be identified by their paths of travel against the drift current. Only cells moving counter to the thermal drift were selected for tracking, as they clearly represented active motility.

**FIGURE 7:**
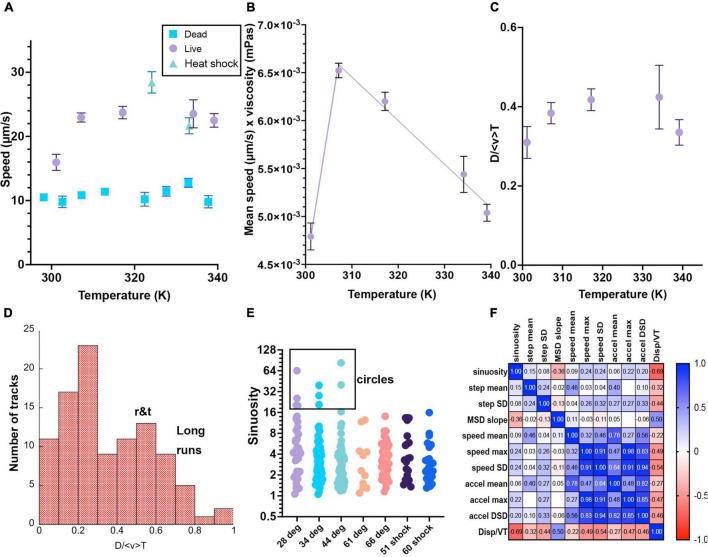
Selected motility parameters. Definitions of the parameters, values and statistical significance are given in the text. Error bars shown are mean ± standard error of the mean unless noted. Numbers of analyzed tracks and their classifications are given in Table 1. When error bars do not appear, they are smaller than the symbols. **(A)** Mean speed. **(B)** Mean speed times viscosity of water at that temperature. The lines are linear fits to temperatures above or below 310 K. **(C)** Total displacement normalized to track length and average speed<v>. **(D)** Histogram of selected values of D/ <v> T, showing classification into long runs vs. run-and-tumble traces. **(E)** Sinuosity (no error bars given as the distributions were not Gaussian and the mean value had little significance). These values are plotted on a Log_2 scale for ease of visualization of the ranges involved. Circular tracks were identified as those with sinuosity >20. **(F)** Correlation matrix of the measured parameters.

The fraction of motile cells (see Table 1 for statistics) increased from ∼20% at 28°C (Figure 6A and Supplementary Video 2) to 25% at 34°C (Figure 6B and Supplementary Video 3) to >60% 44°C (Figure 6C and Supplementary Video 4). However, at 61 and 66°C, the fraction of motile cells was greatly reduced, to <6% and <1%, respectively. Motile tracks could still be identified in single-plane reconstructions (Supplementary Video 5) and in MHI analysis projections through Z (Figures 6D,E and Supplementary Video 6). At 84°C, only thermal currents and drift were observed, with no active counter-current motility; the fraction of motile cells was deemed 0% (Figure 6F and Supplementary Video 7). Qualitative analysis of these tracks on a cell-by-cell basis revealed that there were 4 basic track types: (1) helical tracks (Figure 6G); (2) long straight runs (Figure 6H); (3) circular tracks (spinning) (Figure 6I); and (4) runs with tumbles and directional changes, which could feature either straight or helical swimming or some combination of both; any track with distinct directional changes was classified as run and tumble (Figure 6J).

**TABLE 1:** Motility parameters.

Temperature (°C)	Fraction of cells motile ± SD	Total # tracks analyzed (# replicates)	# fast runs/tumbles/helices/circles	Mean ± SD speed (μm/s) (range)	Mean ± SD acceleration (μm/s^2^)	Mean ± SD displacement (μm/s) (range)
28	20 ± 1	36 (2)	10/8/10/8	16 ± 7 (3–32)	150 ± 70	5 ± 4 (0.2–16)
34	24 ± 2	66 (2)	24/26/8/8	23 ± 6 (12–42)*	200 ± 60*	9 ± 5 (0.7–21)
44	61 ± 2*	57 (2)	18/30/5/4	24 ± 7 (8–45)*	210 ± 90*	10 ± 6 (0.1–25)*
61	6 ± 2*	16 (2)	10/5/1/0	24 ± 7 μm/s (14–38)*	230 ± 90*	10 ± 7 (1.3–27)
66	<1*	35 (2)	18/13/2/0	23 ± 6 (12–41)*	220 ± 70*	8 ± 5 (1.3–26)
51 heat shock	Not done	21 (1)	2/2/10/7	28 ± 6 (15–47)*	250 ± 110*	10 ± 7 (2–24)
60 heat shock	Not done	24 (1)	2/12/8/2	22 ± 6 (13–37)*	160 ± 70	8 ± 5 (1.1–18)

“Replicates” represent independent experiments done on different days with fresh cultures of B. subtilis. Tracks were characterized according to Figure 6 by visual inspection and correlation with parameters as in Figure 7. SD, standard deviation. (*), significantly different from value at 28°C (p < 0.01).

Heat-killed cells showed no active motility, and the MHI images correspondingly showed few features at the lower temperatures (Supplementary Figure 6). Amplitude reconstructions of the holograms revealed cells undergoing slight drift and Brownian motion (Supplementary Video 8 shows 28°C). At temperatures of 60°C and higher, substantial thermal drift and convection became apparent. At the highest temperatures, multiple drift planes could be observed, but in all cases motion was clearly due to bulk flow without any evidence of run and tumble events (Supplementary Figure 6 and Supplementary Video 9 shows 84°C). HELM’s classification algorithm was 100% successful at classifying tracks as non-motile at temperatures <50°C, and >95% successful at higher temperatures (Supplementary Video 10 shows classification of tracks at 84°C). This classification is not based upon any one parameter, and correlating its results with physical parameters will be the subject of a future study. Visual inspection is always required to correlate tracks with cells and to perceive patterns suggesting active motility.

Quantitative analysis of tracks was performed by using the automated tracker combined with the MHI to identify tracks that represented valid cell trajectories. Tracks that were not following organisms or which were fewer than 15 frames long were excluded from analysis. Table 1 gives classification of the analyzed motile tracks for selected datasets. Parameters extracted from these tracks are shown in Table 1 and Figure 7. Data from independent experiments were consistent, so full tracking was performed using selected tracks from pools of replicates (see Supplementary Figure 7 for comparisons of replicate experiments). Plotted in Figure 7 are selected parameters where significant differences were seen among the different temperatures or where parameters assisted in classifying tracks. The full datasets are available in Supplementary Datasheets 2–7.

The **mean speed** was defined for each trajectory as


(5) $$\bar{v}=\frac{1}{N}\sum_{i=i}^{N}\frac{||{\vec{x}}_{i}-{\vec{x}}_{i-1}||}{\Delta t},$$

where *N* is all of the points in the identified trajectory and Δ*t* is the (constant) frame rate. There was a significant increase in mean speed between 28°C and all of the other elevated temperatures. There was a statistically significant difference (*p* < 0.001) for comparison between 28°C and the other temperatures, and not significant between any other pairs. The fastest speeds were seen in the 51°C heat shock sample (*p* < 0.001 for comparison with 28, 35, and 60°C heat shock). The values for the 60°C heat shock were comparable to those at all other elevated temperatures (significantly different from 28 to 51°C heat shock, all others non-significant). Values are given in Table 1 and means with standard errors of the mean are plotted in Figure 7A for both live and killed cells; distributions were Gaussian at each temperature. The speeds of the killed cells were significantly less than those of the live cells, even when drift was significant. There was no significant difference in maximum trajectory speed seen between any pairs of data sets of live cells (not shown; means 80–100 μm/s for maximum instantaneous speeds).

The mean speed times viscosity, which should give an approximate measure of flagellar force as given in Eq. (4), decreased essentially linearly at higher temperatures. This is consistent with denaturation of the proteins as the temperatures rise, representing a typical optimum performance curve with the optimal temperature near 310 K (37°C) (Figure 7B). Because heat denaturation is a time-dependent process involving multiple parameters (Peterson et al., 2007), additional time points at each temperature would be needed to extract meaningful physical parameters from this temperature dependence.

As distinguished from mean speed, **displacement** (μm/s) looks at the whole trajectory rather than each frame, and is the norm of the total XY path, D = $\sqrt{{\left(△x\right)}^{2}+{\left(△y\right)}^{2}}$. For a straight track, displacement will equal mean speed < *v* > multiplied by total time T of the track lifetime; for a circular track, displacement will be close to 0 regardless of mean speed. Dividing the displacement by < *v* > *T* gives a dimensionless number that can be used to classify tracks. Figure 7C shows the average D/ < *v* > *T* vs. temperature, and (Figure 7D) shows a histogram of displacements for selected measured tracks at multiple temperatures; values of D/ < *v* > *T*≥0.55 indicated long runs.

**Sinuosity** is a measure of movement inefficiency defined as end-to-end displacement over total path length in μm. For a straight path, sinuosity will equal 1. For a serpentine or circular movement pattern, sinuosity will be ≫1. Values of >20 were only seen at lower temperatures (Figure 7E). The cell-by-cell classifications in Table 1 agreed with this analysis, as circular tracks were not found in the elevated temperatures.

Acceleration (μm/s^2^) is measured as inter-frame differences in velocity:


(6) $$\bar{a}=\frac{1}{N}\sum_{i=i}^{N}\frac{||{\vec{v}}_{i}-{\vec{v}}_{i-1}||}{△t},$$

and could be used along with mean speed to classify tracks. Long, smooth runs showed low values of acceleration, while tracks with multiple tumble events showed high values. Similarly, the **step angle** measures how much a particle turns at each time point. It gives the angle that a particle deviated from a straight path per inter-frame interval (in radians). Passively drifting particles should not very much in their direction from frame to frame, while highly motile particles that swerve and turn regularly will show large changes in step angle. Figure 7F shows a correlation matrix of the measured parameters, showing that D/ < *v* > *T* showed a negative correlation with nearly all measured values.

**Autocorrelations** of speed, velocity, and turn angle at 1 and 2 s did not vary significantly among datasets (not shown; available in Supplementary Datasheets 2–7).

### Surface Clustering

**FIGURE 8:**
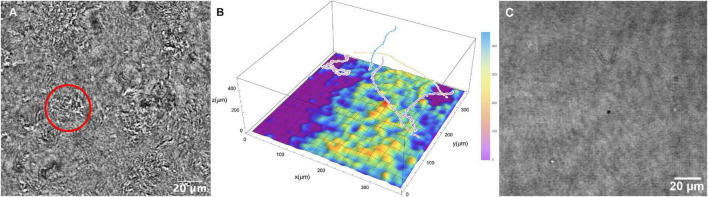
Collections of non-motile cells on the chamber surface at 66°C. **(A)** Still image; the circle indicates a cluster of cells. **(B)** Schematic of surface clustered cells with relative positions of selected motile tracks. **(C)** Corresponding image of the slide surface with the killed cell dataset at 66°C.

At higher temperatures (60°C and above), cells were seen to be swimming above large patches of surface-adherent bacteria. The Z projections could not determine how far the highly motile cells were from the patches, or whether they were interacting. Thus, it was necessary to use full Z plane reconstructions to identify the relative position of motile cells vs. collections of non-motile cells at the chamber bottom. It was seen that the highly motile cells were moving at focal planes tens to hundreds of microns away from the chamber bottom (Figures 8A,B and Supplementary Video 11). While some active motility was seen near the clustered cells at the surface, it was significantly slower as expected near a surface (Li et al., 2008, 2011). Similar groups of cells were not seen with the killed sample (Figure 8C).

## Discussion

The novel immersion system reported in this paper allows for recordings of bacterial motility up to nearly the boiling point of water. The custom software package that we report for the first time here aids in tracking of motile organisms as well as identification of non-motile tracks resulting from drift. Distinguishing drift from motility was straightforward by visual inspection, as active motility could occur perpendicular to the thermal drift. However, the automated algorithm did generate a large number of false positive tracks, so manual validation was essential to accurately identify motile organisms. The MHI images were used to help identify tracks that corresponded to organisms. Although human intervention is needed, this method is significantly easier than manual tracking. As HELM was designed for use on spacecraft, HELM can process a sample in several minutes on an ordinary laptop computer, and manual confirmation of motile tracks may be performed in less than an hour. It is important to note that the current implementation of the software assumes (and checks for) a constant frame rate, so it is necessary to ensure that the acquisition camera and software do not drop frames. Future versions of the software will enable input of a timestamps file to accommodate varying frame rates. An approach to extracting tracks directly from the MHI traces is also in development.

For this work, tracking was only performed on 2D projections of the 3D tracks. The errors introduced in velocities and turn angles by this approximation have been reported (Taute et al., 2015). Given that the axial resolution of the instrument is >2 μm, we decided that in this analysis, the additional computational and time cost of 3D tracking does not contribute significantly to the analysis of velocities, as we have shown in a previous analysis (Acres and Nadeau, 2021). In 2D, helices appear as spirals, with all of the parameters of the helix readily extractable from the 3D data (Gurarie et al., 2011). For organisms or instruments that show higher contrast, HELM could also be used on individual Z planes. However, with our current implementation, the signal to noise of *B. subtilis* is too low for tracking on a single plane, so projections over multiple planes were used. Since most literature data is based upon 2D tracks, this also allows for easier comparison of our measured speeds with those found in other studies. The speeds we observed, 15–25 μm/s, are consistent with the recent reports (Turner et al., 2016). However, large variations from study to study have made general conclusions about parameters as simple as mean velocity for a given strain largely impossible. A new database, named BOSO-Micro (Rodrigues et al., 2021), is aiming to increase standardization of motility experiments and parameters. Such databases will be important for summarizing the large amounts of data being generated in the rapidly emerging field of bacterial tracking.

Using this system and approach, we found that *B. subtilis* was capable of active motility up to 66°C under conditions of constant heating over a time course of 4 h. Cell size and shape did not significantly change with temperature. The changes in speed with temperature were consistent with previous studies up to 50°C (Schneider and Doetsch, 1977), with data unavailable past that point. We observed active motility to 66°C, but with a minority of cells being motile at temperatures above ∼45°C. The majority of cells were found clustered on the lower surface of the chamber at high temperatures. This is consistent with cell population heterogeneity in this species (Kearns and Losick, 2005; Lopez et al., 2009; Syvertsson et al., 2021). In addition, some of the cells in this recording showed a change in the observed motility type, most commonly a change from run-and-tumble to long runs. These parameters were indicated by turn angles, total displacement, and sinuosity in our analysis. Tracks with high sinuosity, indicating circling, were seen only at the lower temperatures. Long runs could be readily identified from total displacement divided by average velocity and time length of the track. The observed effect may result from the highly non-linear viscosity of water at high temperatures, but additional experiments are necessary to attempt to deconvolve viscosity and temperature.

After 66°C, movement in the field was due to thermal drift alone. The thermocouples used to confirm the temperature of the chamber showed a slight difference between the top and bottom glass of the chamber, which could have led to increased thermal currents, most notably at temperatures past the limit for autonomous motion. This drift was clearly distinguishable from active motility on the MHI traces, but attention to correct classification of tracks as motile or non-motile was essential during analysis. The algorithm performed well with the killed samples, identifying nearly all of the tracks as non-motile despite drift, although it did yield a small number of false positives at the highest temperature (84°C). The random forest classifier used to classify tracks as motile or non-motile relies upon numerous parameters, no single one of which is sufficient to classify a track as motile or non-motile. Further analysis will correlate physical parameters with motility; this may require analysis beyond the use of means and standard deviations across an entire track, but close attention to instantaneous parameters. This analysis will also aid in classifying motile tracks with regard to run length and tumble frequency. Custom training of the classification software may be necessary for datasets with substantially different flow profiles.

The MHI traces are a unique feature of HELM, which was designed with spaceflight missions in mind, where the detection of possible signs of life with the least possible processing power is required. The MHI allows visualization of tracks in low signal-to-noise recordings where objects cannot easily be identified by tracking algorithms. These traces may be used as a guide for selection of complete motile tracks identified by HELM. However, compared to other software packages such TrackMate, HELM’s particle identification and tracking is less interactive. When the algorithms do not perform well, the MHI may be used as a guide to finding tracks manually or using other particle detection algorithms in other packages. When HELM’s detection and tracking works well, tracks are exported in.json files which may be imported into other packages for stitching or further analysis.

Reconstruction at individual Z planes at higher temperatures showed rapidly swimming cells tens to hundreds of μm above the surface, with large numbers of cells on the surface, some of which exhibited active motility. The presence of active motility in mesophilic organisms at temperatures beyond those at which they can grow is somewhat of a surprising result. Direct visualization of the motion of individual cells in this work makes this result unambiguous. The use of killed control cells eliminated any possibility that the motion was due to complex thermal currents, and the disappearance of any signs of active motility above 66°C further indicates that the “live” cells became inactive at this point. When reduced viscosity at these temperatures was considered, there was a temperature-dependent reduction in flagellar force. This is likely due to protein denaturation, and longer incubation times at these elevated temperatures may eventually lead to complete loss of motility. More detailed experiments with controlled incubation times at specific temperatures may provide useful models of thermal stability of motility-related proteins (Daniel and Danson, 2010). This paper represents the first step toward evaluating the upper limits of temperature on mesophile motility.

The authors hope that this simple setup will encourage others to reproduce these experiments and examine other strains of bacteria and archaea. In contrast to bacteria, especially test strains such as *E. coli* and *B. subtilis*, hyperthemophilic archaea have not been frequently imaged. The setup we report here should facilitate studies of thermophilic organisms, including those such as *Pyrococcus furiosus* which require temperatures near the boiling point of water for optimal motility (Herzog and Wirth, 2012).

The detailed parts list, combined with the open-source software, should be sufficient to enable duplication of the system by anyone who wishes to perform these experiments. The only custom parts are a 3D printed stage and objective lens holder (plastic) and a custom machined stage (aluminum) to allow for easy changing of sample chambers without requiring stage realignment. CAD drawings of these can be provided on request, and users are encouraged to tailor designs to their own specific applications. It is important to ensure that these elements are made from materials that can withstand high temperatures; any materials chosen should ideally be tested beforehand by submerging them into hot water at the desired temperatures before use on the microscope.

## Data Availability Statement

The raw holograms for the datasets used here are deposited in a public depository at Data Dryad, accession https://doi.org/10.5061/dryad.ns1rn8pv6. Other data are available from the authors upon request. The software packages used are all open source and are available at the following sites: DHMx: https://github.com/dhm-org/dhm_suite; HELM: https://github.com/JPLMLIA/OWLS-Autonomy; and Reconstruction Fiji plug-ins: https://github.com/sudgy/.

## Author Contributions

MD and NJ: construction and calibration of thermal control apparatus, growth and maintenance of bacteria, data collection, data analysis, data archiving, and writing. MW and JL: development of HELM tracking software, troubleshooting and debugging of software, and addition of new features upon request. CL: original concept design and acquisition of funding. JN: acquisition of funding, supervision and planning of experiments, data analysis, and writing. All authors: editing and approval of final draft.

## Conflict of Interest

The authors declare that the research was conducted in the absence of any commercial or financial relationships that could be construed as a potential conflict of interest.

## Publisher’s Note

All claims expressed in this article are solely those of the authors and do not necessarily represent those of their affiliated organizations, or those of the publisher, the editors and the reviewers. Any product that may be evaluated in this article, or claim that may be made by its manufacturer, is not guaranteed or endorsed by the publisher.

## Footnotes

1
https://github.com/dhm-org/dhm_suite

2
https://github.com/sudgy/

3
https://imagej.net/imaging/particle-analysis

4
https://github.com/JPLMLIA/OWLS-Autonomy
