# Supplementary material for: Quantification of Motility in Bacillus subtilis at Temperatures Up to 84°C Using a Submersible Volumetric Microscope and Automated Tracking
Source: Front Microbiol. 2022 Apr 21;13:836808. doi: 10.3389/fmicb.2022.836808 (PMC9069135; doi:10.3389/fmicb.2022.836808)
Supplement: Supplementary file 1 [file Presentation_1.PDF]

Supplementary Material

1 Supplementary Methods

1.1 System Assembly and Use

The parts used in construction of this system are listed in **Supplementary Datasheet 1**. Complete and detailed microscope assembly can be found in U.S. Patent US20160131882A1 and in the following reference: Wallace, J.K., Rider, S., Serabyn, E., Kuhn, J., Liewer, K., Deming, J., Showalter, G., Lindensmith, C., and Nadeau, J. (2015). Robust, compact implementation of an off-axis digital holographic microscope. *Optics Express* 23**,** 17367-17378. doi: 10.1364/oe.23.017367. This section outlines the assembly and use of the microscope and heat bath system. Items in this section are referenced by the 'ref number' in **Supplementary Datasheet 1**.

After microscope assembly, the system must be prepared for submersion and exposure to increasing temperatures, so several precautions must be taken. Previous iterations of the experimental design led to water infiltrating the microscope components, non-viable fiber optic cables, and condensation on the surface of the optics and sample chambers.

First, a housing around the collimator and up to the microscope stage was necessary. It was found that water pressure would push the bags surrounding the microscope into the space between the microscope stage and collimator, blocking the light from the laser. This was solved by custom fitting the Newark Minibox metal enclosure (42) to the microscope. The box was assembled, and one side was removed to serve as the top, which sits just below the microscope stage. On one of the sides adjacent to the removed top, notches were cut so the box slid up around the arms holding the collimator to the base of the microscope. For this setup, the notches are shown in **Figure S1**. This allows the enclosure to slide parallel to the base of the microscope and sit just below the stage. It is important to check that the enclosure is long enough to: a) cover the gap between the collimator and microscope stage, and b) give the fiber optic cable enough room to loosely sit below the collimator and curve to exit out the top of the enclosure without bending or warping the cable.

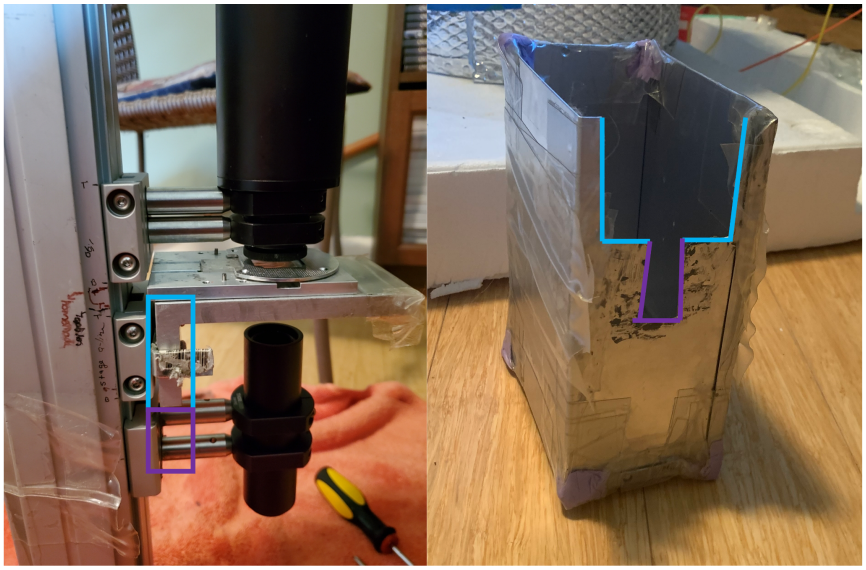

Figure S1. Notches cut into metal enclosure to fit around base of the microscope stage and the collimator attachment. Metal enclosure should sit slightly below the stage when attached.

Once the notches have been cut in the enclosure, any sharp edges on the enclosure need to be covered to prevent tearing of the bags. Any substance which covers the sharp edges is fine, but it should be one that will not degrade with exposure to high temperatures. In this case, torn nitrile lab gloves were layered around the corners to act as a bumper and then held in place with heat-resistant carpet tape. This was done in layers to ensure any sharp edges were covered. The heat-resistant tape was then put along all sides of the enclosure, even the machined edges. The tape was then used to secure the enclosure to the base of the microscope. The enclosure should be adhered in a way that is stable enough to withstand the experiment, but also allow for easy removal in case of accidents or maintenance.

Once the enclosure is attached to the microscope, the system is ready for submersion. First, place the sample chamber on the stage and make sure it is fixed in place. Take some sample recordings to check the focus since it cannot be adjusted later without removing the microscope from the heat bath. The Ziploc bags (41) are recommended. They were able to withstand boiling without degradation and breaking. In initial iterations of the experiment, autoclave bags were used. These are less durable but do provide a more flexible barrier than the Ziploc bags. If using autoclave bags, the bags should definitely be double layered around the microscope, since degradation after prolonged exposure was an issue. Fill the majority of the pot (40) with water before placing the microscope in. Submerge the microscope and bags until the bottom of the base is at the bottom of the pot. Using an F-clamp, clamp the base of the microscope to the outside of the pot. After the clamp is tightened, check to be sure the stage of the microscope is at a level that is still within the pot. Attach the sous vide (39) to the opposite side of the pot and fill the pot with water until the level is at least 1” above the microscope stage. Insert dowel-like objects (kitchen spatulas/spoons work just fine) into the space between the metal enclosure and the stage, and stick out of the top of the bags and pot. This is to prevent condensation from forming on the surface of the sample chamber or any of the optics.

The pot should be placed on an insulating surface, since heat loss from the pot can reach the same order of magnitude as the rate at which the sous vide heats the water. This was noted primarily at temperatures >~66C. In this setup, a Styrofoam block several inches thick was used. To insulate the sides, wrap the exterior of the pot in insulating material (metallized bubble wrap bags from Imperfect Foods worked well when cut to form a single long strip). Wrap the material tightly and adhere with heat-resistant adhesive. The pot will be very hot as the experiment progresses, so having a tape that will withstand that is important. Carpet tape is a good option. Lastly, cut insulating material to form a cover for the bath. The imperfect food bag can be cut along the seam until it resembles a tent with flaps on one side. Place the "closed" end of the tent over the top of the pot at the side with the sous vide, so the flaps wrap around the column of the microscope. Make sure not to put the insulating material over the camera, as condensation will occur. This is to keep heat in from the top of the pot, critical for temps >~78C. The stages of wrapping the pot and submerging the microscope are shown in **Figure S2**.

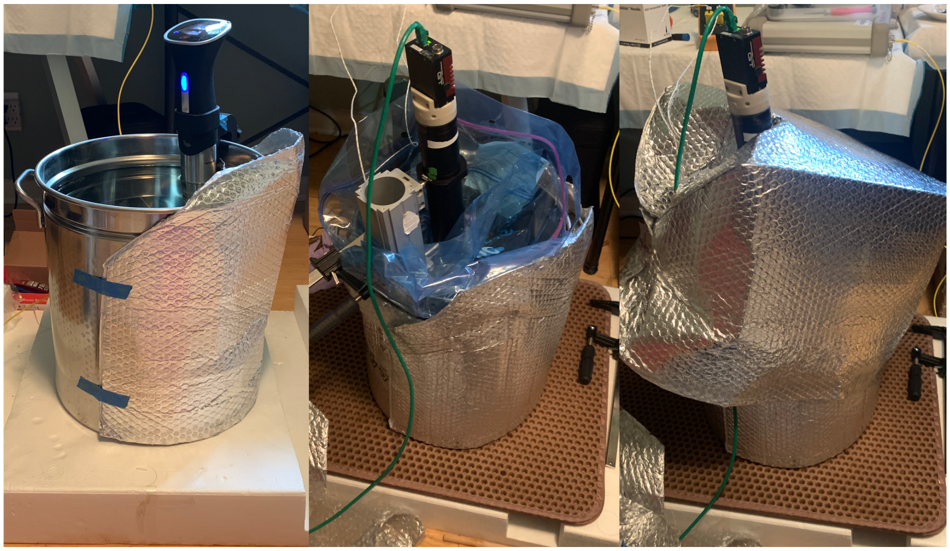

Figure S2. Progression of insulating the pot and submerging the microscope, from left to right.

1.2 Sample chambers

The sample chambers used in the experiment were custom designed, as referenced in the methods section. However, this system was also tested with sample chambers that are simpler and can be made quickly in the laboratory. This section outlines the construction of simple, effective sample chambers that have been tested and are suitable for use at the range of temperatures described. They were made to contain both a sample and reference channel that are filled separately. They consist of optically clear glass tops and bottoms, with a gas-permeable layer giving depth to the chamber, as shown in **Figure S3**.

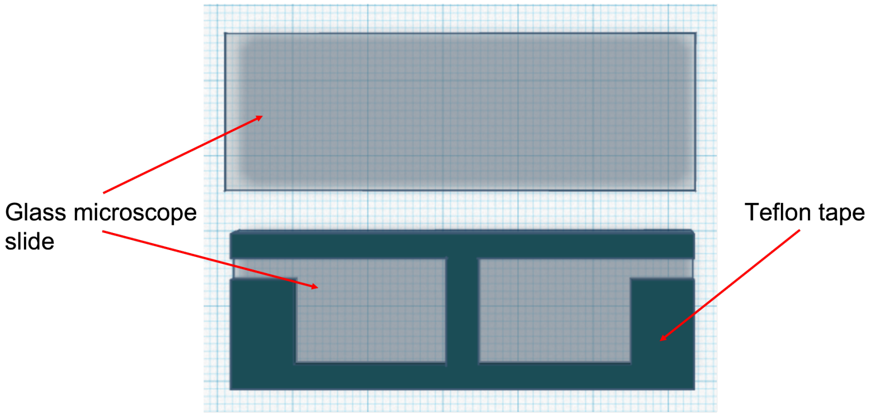

Figure S3. Model of the component pieces of a simple, custom sample chamber for DHM. The glass slides form the top and bottom of the chamber and the teflon tape is cut to form the sample and reference wells. The chamber depth is 0.5mm.

The chambers are made from two 25mm x 75mm x 1mm glass microscope slides (Fisherbrand or other) and a piece of 0.5mm thick Teflon tape (CS Hyde, Item #: 15-20HB-DS-1-5, .020" Skived PTFE w/ High Bond) cut to the same dimensions as the microscope slides. The Teflon is then cut in the shape shown (**Figures S3,S4**), with the central barrier ~4mm wide. For the instrument used in this experiment and described in Supplementary Datasheet 1, the separation of the objective lenses is 6.0 mm, so this middle barrier should not exceed this distance. A channel was also cut to the edges of the Teflon for the wells to be filled once the chamber was completely assembled.

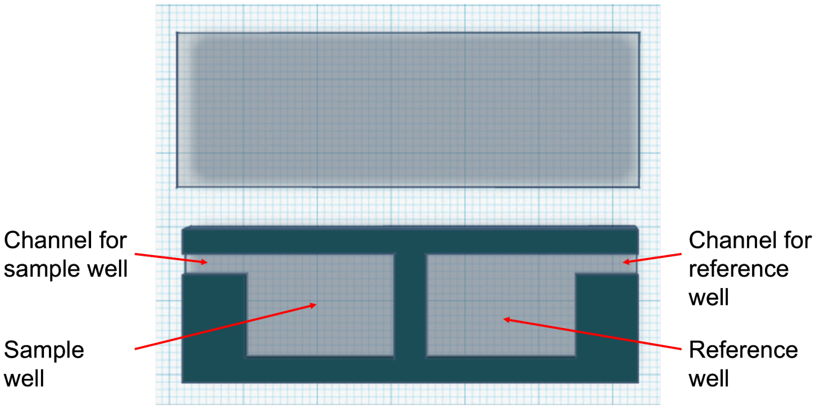

Figure S4. Modeled setup of sample chamber with separate sample and reference wells. Designed with channels for filling after chamber construction to prevent bubbles. Middle barrier separating the wells should be thinner than the separation of the objective lenses of the microscope. For the microscope used here, that is less than 6.0mm.

Adhesive was placed along the bottom side of the Teflon to adhere it to one of the microscope slides. Once the adhesive is set, apply adhesive to the top side of the Teflon and adhere the other microscope slide. Dow Corning high vacuum grease can also be used, but is less stable because it does not solidify. Allow to sit while the adhesive cures. The completed setup is shown (**Figure S5**).

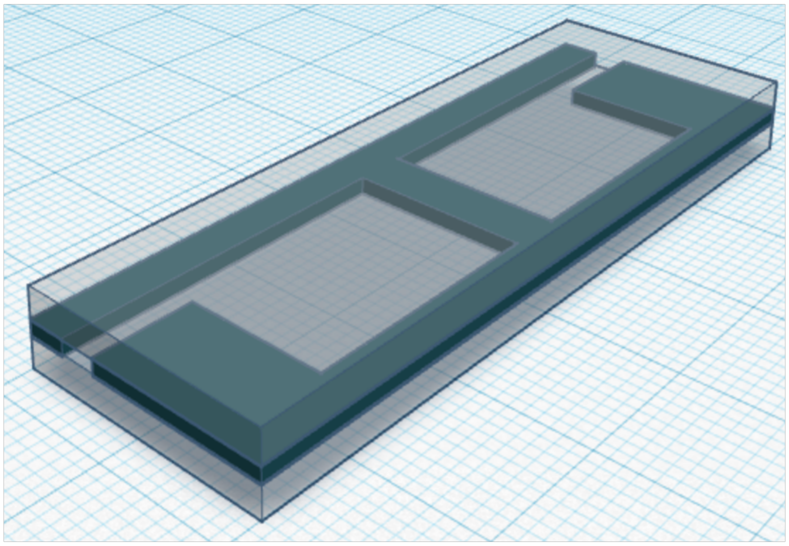

Figure S5. Model of the completed chamber described in this section. Gaps from the channel should be filled with sealant or vacuum grease to prevent leaking and bubbles.

When ready for use, inject the specimen into the sample well using a needle. Once the well is filled, seal the channel. Silicone sealant or high vacuum grease recommended. Repeat these steps with the reference well and adhere the chamber to the stage before proceeding.

2 Supplementary Data

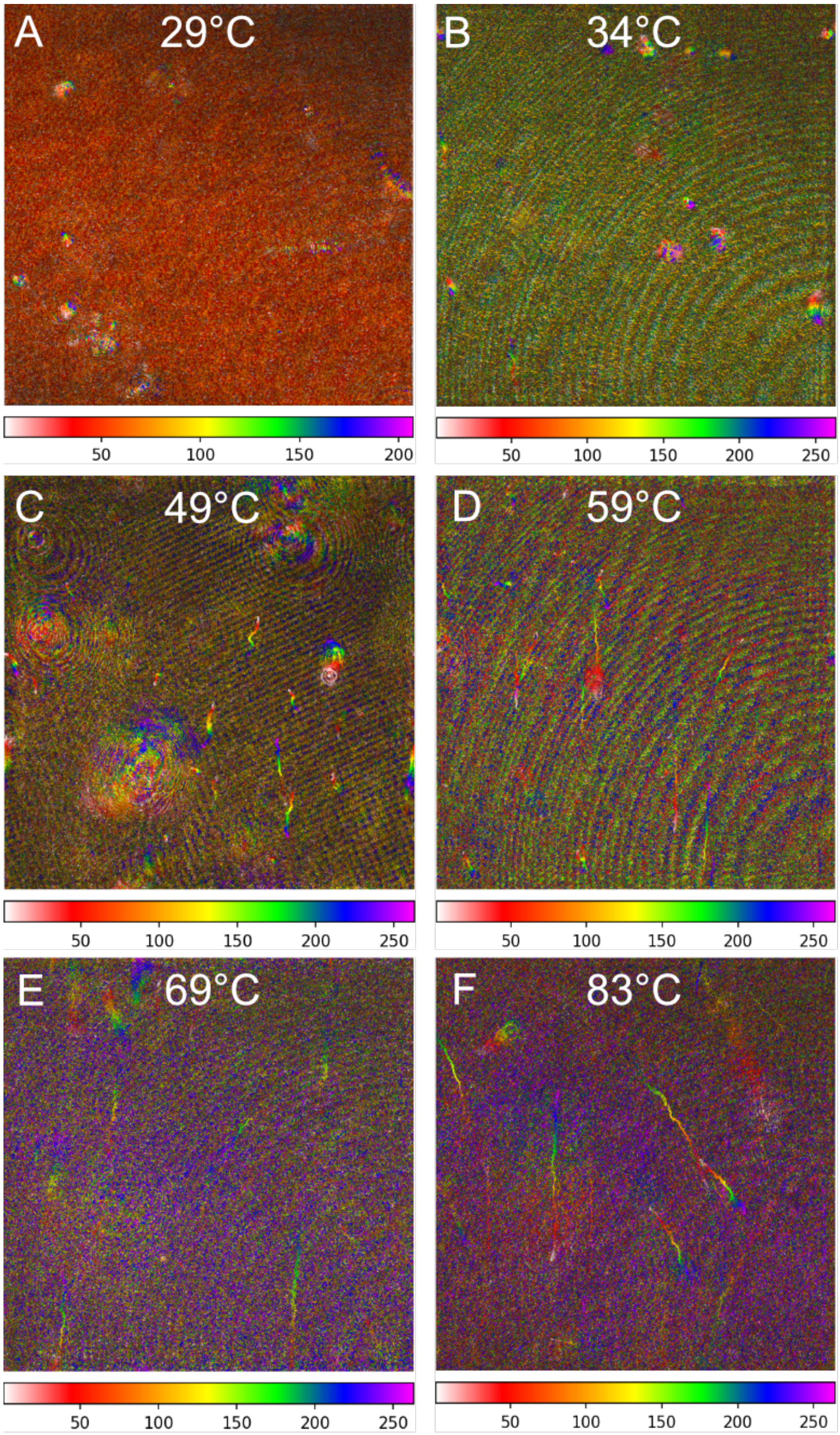

Figure S6. MHI tracks from killed *B. subtilis* at different temperatures. The field of view is 365 x 365 µm and the color scales indicate time in frames (15 frames/s).

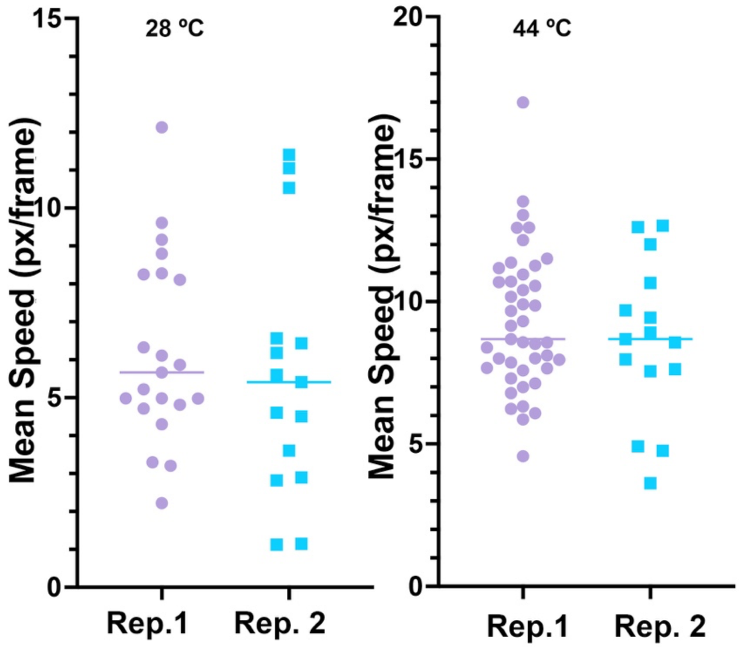

Figure S7. Consistency between replicate experiments. Examples of mean speeds from 2 replicates done on independent days, showing consistency of values.

Video captions

Supplementary Video 1. Example of automated tracker using a 34 ºC data set. Tracks in cyan are identified by the software as non-motile, and those in magenta as motile. Tracks were handpicked and re-classified as necessary, using the MHI as a guide in order to match identified tracks to organisms.

Supplementary Video 2. Portion of a single plane reconstruction at 28 ºC. 250 frames at 15 frames/s.

Supplementary Video 3. Portion of a single plane reconstruction at 34 ºC. 250 frames at 15 frames/s.

Supplementary Video 4. Portion of a single plane reconstruction at 44 ºC. 250 frames at 15 frames/s.

Supplementary Video 5. Portion of a reconstruction 180 µm above the plane of the slide at 61 ºC. 250 frames at 15 frames/s.

Supplementary Video 6. Maximum z-projection at 66 ºC. 250 frames at 15 frames/s.

Supplementary Video 7. Portion of a single plane reconstruction at 84 ºC. 250 frames at 15 frames/s.

Supplementary Video 8. Killed cells, single plane reconstruction at 28 ºC. 260 frames at 15 frames/s.

Supplementary Video 9. Killed cells, single plane reconstruction at 84 ºC. 260 frames at 15 frames/s.

Supplementary Video 10. Results of HELM tracker and classifier with killed cells at 84 ºC. 260 frames at 15 frames/s. Tracks in cyan are identified by the software as non-motile, and those in magenta as motile.

Supplementary Video 11. Portion of a reconstruction at the plane of the slide at 61 ºC. 250 frames at 15 frames/s.
